# Assessment of heavy metal sources and health risks in soil-crop systems of fragmented farmland

**DOI:** 10.3389/fpubh.2025.1637595

**Published:** 2025-07-31

**Authors:** Lei Tong, Aoran Yang, Mingyue Fan, Daikun Zheng, Ji Li, Hanlin Liu, Chenggen Li, Yongqiong Tang, Longfeng Cheng

**Affiliations:** ^1^School of Public Health and Management, Chongqing Three Gorges Medical College, Chongqing, China; ^2^Department of Environment and Food Hygiene, Chongqing Wanzhou District Center for Disease Control and Prevention, Chongqing, China

**Keywords:** heavy metals, crops, IICQ index, Monte Carlo simulation, health risks

## Abstract

Heavy metal contamination in agricultural ecosystems poses significant risks to human health. Metals accumulating in soil-crop systems can be absorbed and concentrated by crops. Chronic exposure through the consumption of contaminated crops may lead to severe health consequences. This study investigated eight common heavy metals (Cd, Ni, As, Cu, Hg, Pb, Cr, Zn) in the soil-crop system of Wanzhou District, Chongqing City, and performed a probabilistic risk analysis using Monte Carlo simulation. The study results show that for non-carcinogenic risks, the total hazard quotient for adults (2.365) and children (1.176) both exceed the safety threshold of 1.0, with As contributing significantly to population health risk. For carcinogenic risks, the total carcinogenic risk for adults (2.28 × 10^−^3) and children (1.11 × 10^−^3) significantly surpass the unacceptable risk level of 1.0 × 10^−^4, with As, Cr, Cd, and Ni presenting particularly high carcinogenic risks to the population. Additionally, the sensitivity analysis revealed that the concentration (C) of heavy metals in crops is a key exposure parameter influencing the health risks for both adults and children. Given the significant contributions of As, Cr, Cd, and Ni to health risks, these metals should be prioritized for monitoring and control. Long-term intake of crops with excessive heavy metal content increases health risks, highlighting the urgent need to address heavy metal pollution in urban fragmented farmland soil-crop systems.

## 1 Introduction

Rapid urbanization and industrialization have made soil heavy metal (HM) pollution an important environmental issue. Soil pollution from HM is characterized by uneven distribution, easy accumulation, non-biodegradability, and low volatility, which lead to the long-term persistence of HM pollutants in the external environment (1, 2). Data from the national communique of soil pollution survey by the Ministry of Environmental Protection of China and the Ministry of Land and Resources in 2014 revealed that the exceedance rate of HMs in arable land soil in China was 19.4%, and HMs were the main pollutants. The exceedance rates for cadmium (Cd), nickel (Ni), arsenic (As), copper (Cu), mercury (Hg), lead (Pb), chromium (Cr), and zinc (Zn) were 7.0, 4.8, 2.7, 2.1, 1.6, 1.5, 1.1, and 0.9%, respectively (3). HM pollution not only deteriorates soil quality and reduces crop yields but also poses a serious threat to human health through bioaccumulation and the food chain (4–7).

HM exposure in the human body mainly occurs through three routes: the respiratory tract, digestive tract, and skin, with dietary intake through the digestive tract accounting for over 90% of the total exposure (8, 9). Long-term consumption of crops contaminated with HMs can adversely affect various human systems, including the nervous, skeletal, circulatory, and immune systems (10, 11). It can also alter enzymatic activity, disrupt hormone secretion, and cause cancer (12–14). Various crops provide the energy, protein, and nutrients required for human physiological activities, and their quality and safety are directly related to human health. In some Asian and African regions, there are similar severe HM pollution problems like those in our study area. For example, in Pakistan, studies on drinking water, soil, and vegetables have found that in some local areas, the Cd, Cr, and Ni content in drinking water is above safe limits. Also, vegetables are contaminated with Cr, Ni, Cd, and Pb. Long-term consumption of these vegetables can increase health risks, especially for children (15–17). A study in East Cameroon shows that the average content of Fe, Al, Cr, Mn, Ba, Zn, Pb, Ni, Cu, Co, As, and Se (mg/kg) is higher than the FAO–set safety limits. The carcinogenic risk index indicates that Cr has the highest cancer risk, with children being more vulnerable than adults (18). Studies in India and Bangladesh also show similar HM pollution problems (19–22). This demonstrates the global impact of HM pollution and its serious burden on public health. In China, relevant studies also indicate the severity of heavy metal pollution. For instance, a study conducted in Wenling City, Zhejiang, showed severe Cd contamination in rice, with Cd, Cr, and As being the chief contributors to its overall carcinogenic risk (23). Similarly, a study in Shanghai found contamination with Pb, Cd, and As in vegetables (24). Furthermore, an analysis of HM pollution in vegetables from urban and suburban areas in China revealed numerous samples with excessive levels of Cd, Hg, and Pb in urban and suburban vegetables, indicating the seriousness of HM pollution (25). Economic development and the improvement of living standards have increased the variety and quantity of various crops consumed and the risk of HM exposure from crops (26).

The assessment of soil environmental quality typically focuses on HMs in the soil, and methods used for evaluation include the single pollution index, Nemerow's comprehensive pollution index, geo-accumulation index, potential ecological risk index, and target hazard quotient index (27–30). However, these methods mainly rely on background values of HMs and screening values, focusing more on the pollution status of individual or multiple HMs in the soil (31, 32), and are unable to conduct a comprehensive pollution assessment within the soil-crop system. In practice, HM pollution often involves both soil and crops, necessitating paired sampling of soil and crops and the use of integrated methods to assess the environmental risks of HMs. This involves considering the mutual influence between HMs in soil and agricultural products on the environmental quality of farmland soil. The Impact Index of Comprehensive Quality (IICQ), comprising the Impact Index of Comprehensive Quality in soil (IICQ_s_) and the Impact Index Comprehensive Quality in agricultural products (IICQ_ap_), enables a comprehensive evaluation of soil environmental quality and agricultural product safety related to HMs (33). This approach has been successfully used to assess the HM pollution situation in the soil-crop system (34–36).

Wanzhou District in Chongqing City is an ecologically sensitive region and lies at the heart of the Three Gorges Reservoir (37). The rapid development of urbanization and industrialization around the Three Gorges Reservoir has significantly increased the discharge of industrial and urban wastewater, along with the emission of other pollutants. Elevations in the amount of pollutant emissions facilitate the entry and accumulation of HMs in agricultural soils, posing significant risks to local agriculture and public health (38, 39). Wanzhou District is a typical karst region characterized by a high geological background. Carbonate rocks serve as an important reservoir for HM elements, which can be enriched in the soil through weathering and enter the human body through the food chain, posing a threat to human health (40). Thus, we speculate that this region may experience heavy metal pollution, potentially impacting local population health via the soil–crop system. This study investigates HM pollution in soils and crops in the fragmented farmlands of Wanzhou District, Chongqing, and their potential risks to local food safety. The objectives of this study are: (1) to determine the pollution levels and spatial distribution of HMs in Wanzhou District; (2) to analyze the levels of HM pollution in the soil-crop system; (3) to explore the possible sources of HMs using correlation analysis and Mantel test analysis; (4) to assess the potential health risks of HMs in crops to adults and children. This study enhances our understanding of heavy metal pollution in local soils and crops, as well as the associated health risks. The research results not only help evaluate the heavy metal environmental exposure level, but also provide a scientific basis for subsequent policymaking. Through this study, we aim to reduce the risk of heavy metal environmental exposure and minimize the health hazards caused by the pollution.

## 2 Materials and methods

### 2.1 Study area and sample collection

**Figure 1:**
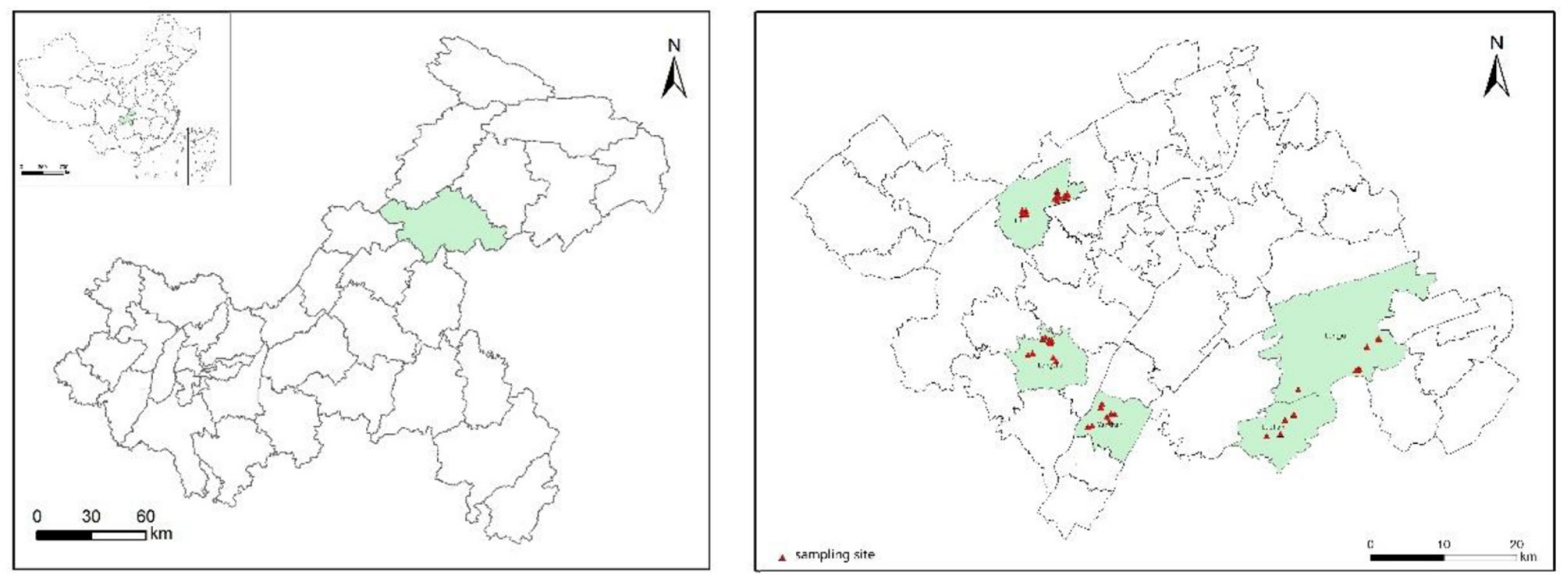
Distribution of sampling sites for soil and crops samples.

The study area is located in Wanzhou District (30°3′50″-310′18″N, 107°2′22″-108°3′52″E), in the northeastern part of Chongqing, at the heart of the Three Gorges Reservoir region. The study area has an approximate area of 3,456.41 km^2^, with an administrative division that includes 14 sub-districts, 27 towns, and 11 townships, and a total population of roughly 1.55 million people. The district experiences a subtropical monsoon climate, with distinct seasons, an average annual temperature of 15–24°C, and an average annual precipitation of 1,000–1,200 mm, with the flood season concentrated from May to September. By 2022, the region had a total agricultural acreage of 83,908 hectares, with paddy fields accounting for 30,622 hectares (36.49%) and dry land making up 53,286 hectares (63.51%). The main soil types include purplish soil, paddy soil, and yellow soil. The research focused on fragmented cropland in the district. Based on preliminary soil monitoring data, the sampling sites covered five representative towns: Lihe, Longju, Longsha, Yanshan, and Luotian town. In 2023, 50 crop samples, including four crop types (26 grains, nine vegetables, three leaf vegetables, 12 tubers) and their rhizosphere soil samples, were collected from the study area. Figure 1 shows the distribution of sampling spots. Soil sample collection was conducted in accordance with the “Soil Environmental Monitoring Technical Specifications” (HJ/T166-2004) (41). Soil samples were collected from a depth range of 5–20 cm using the plum blossom grid method to ensure uniform sampling over an area of 1 m^2^. Subsampling was performed using the quartering method, retaining 1 kg of soil per sample. Additionally, we also collected samples of the corresponding crops grown on the soil. After mixing, we used the quartering method for subsampling, retaining 500 g as one sample, and properly recorded the sampling process.

### 2.2 Sample testing analysis, methods, and quality control

In the laboratory, soil samples were dried, ground, and sieved through a 100-mesh sieve. Subsequently, an HCl-HNO_3_-HF-HClO_4_ solution was added, and the samples were heated at 150°C for 48 h to perform digestion (42–45). Grain samples were rinsed with purified water, dried, threshed, dehulled, and ground to a 40-mesh size and then microwave-digested to determine the concentration of HMs. Vegetable, leaf vegetable, and tuber samples were cleaned with purified water, homogenized, and microwave-digested to determine the concentration of HMs. A programmed temperature ramp was applied to gradually increase the temperature to 185°C, followed by 40-min microwave digestion. The soil pH value was determined using the potentiometric method as specified in the NY/T 1377-2007 (46). The Hg content in soil was measured using the catalytic thermal decomposition-cold atomic absorption spectrophotometry method (HJ 923-2017) (47). The concentration of HMs such as Pb, Cd, Cr, As, Cu, Zn, and Ni in soil were measured using the aqua regia extraction-inductively coupled plasma mass spectrometry (ICP-MS) method (HJ 803-2016) (48). The presence of Hg in crops was assessed using direct mercury measurement (Method II of GB 5009.17-2021) (49), while Pb, Cd, Cr, As, Cu, Zn, and Ni were measured using the inductively coupled plasma mass spectrometry (ICP-MS) method (Method I of GB 5009.268-2016) (50).

Soil and crop detection indicators both adopt standard methods. Instruments used are the Inductively Coupled Plasma Mass Spectrometer NexION 2000 (PerkinElmer, USA), the Fully Automatic Direct Mercury Analyzer MAX-S (Leiberte), and the Multi–parameter Tester pH Meter (Mettler–Toledo). The main reagents used are nitric acid [Thermo Fisher Scientific (China) Co., Ltd.] and hydrochloric acid (Chengdu Kelong Chemicals Co., Ltd., chemically pure). All instruments are verified/calibrated, and all standards are within the validity period.

For quality assurance (QA) and quality control (QC), all sampling and testing personnel were uniformly trained, all equipment was calibrated and verified, all standard materials were within their valid period, and quality control measures such as laboratory blanks, parallel samples, and recovery tests were employed during testing. The results of all quality control measures were within the acceptable range.

### 2.3 Evaluation methods

#### 2.3.1 Index of geoaccunulation

The geoaccumulation index (*I*_geo_) was introduced in the early 1960s to assess the elemental contamination level in sediment. *I*_geo_ values were calculated using the following equation (51, 52):


(1) $$\begin{array}{l} I_{geo}=\log_{2}\left(C_{soil}/kC_{bi}\right) \end{array}$$

where *C*_soil_ is the measured HM concentration in the sample, *C*_bi_ is the average HM concentration in the study area, *k* is the empirical coefficient, which, in general, is 1.5.

The *I*_geo_ can be classified into different grades: Class 0, *I*_geo_ ≤ 0, uncontaminated; Class 1, 0 < *I*_geo_ ≤ 1, mildly contaminated; Class 2, 1 < *I*_geo_ ≤ 2, moderately contaminated; Class 3, 2 < *I*_geo_ ≤ 3, moderately to heavily contaminated; Class 4, 3 < *I*_geo_ ≤ 4, heavily contaminated; Class 5, 4 < *I*_geo_ ≤ 5, heavily contaminated to extremely contaminated; Class 6, *I*_geo_ ≥ 5, extremely contaminated.

#### 2.3.2 Bioaccumulation factor

The bioaccumulation factor (BCF) reflects the migration characteristics of HMs with soil-crop systems and can be calculated using the following equation (53, 54):


(2) $$\begin{array}{l} BCF=C_{api}/C_{soil} \end{array}$$

where *C*_api_ is the HM contents in the edible parts of crops, and *C*_soil_ denotes HM content in the rhizosphere soil samples.

#### 2.3.3 IICQ index

The Impact Index of Comprehensive Quality (IICQ) was used to assess the relative effects of HM contamination on soil quality at particular sites under individual or composite pollution conditions. Compared to previous assessment methods, IICQ is more scientific and rational as it incorporates HM background values, HM screening values in soil, and permissible limits of HM contents in crops. This approach allows for the simultaneous consideration of soil environmental quality and crop quality, facilitating a quantitative and comprehensive estimation of HMs in soil-crop systems. Furthermore, IICQ can help compare regional soil environmental quality as it can be easily visualized on maps at different scales. In this study, the IICQ of soil-crop systems included the Impact Index of Comprehensive Quality in soil (IICQ_s_) and the Impact Index Comprehensive Quality in agricultural products (IICQ_ap_). These indices were calculated as follows (33, 53, 55):

(1) Relative impact equivalent (RIE) in soil:

(3) $$\begin{array}{l} RIE=\left[\overset{N}{\underset{i=1}{\sum }}{\left(P_{ssi}\right)}^{1/n}\right]/N=\left[\overset{N}{\underset{i=1}{\sum }}{\left(C_{soil}/C_{si}\right)}^{1/n}\right]/N \end{array}$$

Where *P*_ssi_ is the single pollution index of element *I*; *N* is the number of elements; *C*_soil_ and *C*_si_ are the content and screening value of element I, respectively; *n* is the oxidation number of element i (Pb,2; Hg,2; As,5; Cr,3; Cd,2; Cu,2; Ni,2; Zn,2).

(2) The degree of deviation between detected and background value (DDDB) in soil:

(4) $$DDDB=[\sum_{i=1}^{N}{\left(P_{sbi}\right)}^{1/n\;}]/N=\;[\sum_{i=1}^{N}{\left(C_{soil}/C_{bi}\right)}^{1/n\;}]/N\;$$

where *P*_sbi_ is the ratio of HM measured value to its background value in soil for element *i*, and *C*_bi_ is the background value of element *i*.

(3) The degree of deviation of the soil environmental quality standard from the background value (DDSB):

(5) $$\begin{array}{l} DDSB=\left[\overset{N}{\underset{i=1}{\sum }}{\left(C_{si}/C_{bi}\right)}^{1/n}\right]/N \end{array}$$

Where the symbols correspond with those in the equations mentioned above.

(4) The quality index of agricultural products (QIAP):

(6) $$\begin{array}{l} QIAP=\left[\overset{N}{\underset{i=1}{\sum }}{\left(P_{api}\right)}^{1/n}\right]/N=\left[\overset{N}{\underset{i=1}{\sum }}{\left(C_{api}/C_{isi}\right)}^{1/n}\right]/N \end{array}$$

where *P*_api_ is the ratio of HM measured value to its threshold limit value of crops for element *i* and *C*_api_ is the measured content of element *i* in crops, and *C*_isi_ is the threshold limit value of element *i* in crops.

(5) The impact index of comprehensive quality (IICQ):

(7) $$\begin{array}{l} IICQ_{s}=X\cdot \left(1+RIE\right)+Y\cdot \left(DDDB/DDSB\right) \end{array}$$

(8) $$\begin{array}{l} IICQ_{ap}=Z\cdot \left(1+QIAP/k\right)+QIAP/k\cdot DDSB \end{array}$$

(9) $$\begin{array}{l} IICQ=IICQ_{s}+IICQ_{ap} \end{array}$$

where IICQ comprises IICQ_s_ and IICQ_ap_, which are the respective impact indices for soil and agricultural products. *X* and *Y* denote the number of soil measurements exceeding the soil standard values and soil background values, respectively. Meanwhile, *Z* denotes the number of agricultural products exceeding the pollutant limit standards; *k* is the factor of background correction, commonly set as five.

Based on IICQ_s_, IICQ_ap_, and IICQ, the HM pollution risk was classified into five grades as follows: clean (IICQ ≤ 1), slight pollution (1 < IICQ ≤ 2), light pollution (2 < IICQ ≤ 3), moderate pollution (3 < IICQ ≤ 5), and heavy pollution (IICQ > 5).

### 2.4 Health risk assessment

Health risk assessment method proposed by the United States Environmental Protection Agency can be used to assess the health risks posed by HMs. Since the carcinogenic and non-carcinogenic effects caused by environmental factors are different in risk characterization, we divided them into carcinogenic risk and non-carcinogenic risk when conducting quantitative risk estimation. The hazard quotient (HQ) and cancer-risk index (CR) were adopted to evaluate the potential non-carcinogenic risks and carcinogenic risks from HMs in crops, respectively. Multiple HMs may have a synergistic effect, so the total hazard quotient (THQ) and total carcinogenic risk (TCR) of crops were determined based on the sum of the HQ and CR, respectively. These were calculated through the following equation (56–58):


(10) $$\begin{array}{l} \text{HQ}=\left[\left({\text{C}}_{\text{api}}\times IR\times EF\times ED\right)/\left(BW\times AT\right)\right]/RfD \end{array}$$

(11) $$\begin{array}{l} CR=\left[\left({\text{C}}_{\text{api}}\times IR\times EF\times ED\right)/\left(BW\times AT\right)\right]\times SF \end{array}$$

(12) $$\begin{array}{l} THQ=\sum HQ_{i} \end{array}$$

(13) $$\begin{array}{l} TCR=\sum CR_{i} \end{array}$$

where *C*_api_ was the HM content in crops (mg·kg^−1^), IR was the ingestion rate of crops (kg·day^−1^), EF was the exposure frequency (day·year^−1^), ED was the exposure duration (year), BW was the body weight (kg), AT was the average exposure time for carcinogenic (non-carcinogenic) effects (day), RfD (oral reference dose) was the corresponding reference toxicity threshold dose (mg·kg^−1^·day^−1^), and SF was slope factors (kg·day·mg^−1^).

HQ and THQ values < 1.0 were considered safe, but values exceeding 1.0 suggested potential adverse effects on human health. CR and TCR values above 1.0 × 10^−4^ indicated unacceptable risks, values below 1.0 × 10^−6^ implied no obvious hazard, and values between 1.0 × 10^−6^ and 1.0 × 10^−4^ were acceptable. In order to avoid the uncertainties from using single-point deterministic parameters, which may result in either overestimation or underestimation of risks, Monte Carlo simulation with 10,000 iterations was employed to evaluate the population health risk. The probability distributions and values for the mentioned parameters are detailed in Supplementary Table S1.

### 2.5 Statistical analysis

All spatial distribution maps were generated using ArcGIS 10.2. Statistical analyses were performed using Excel 2016 and IBM SPSS 27. RStudio and Origin 2024 were used for data visualization. Additionally, Monte Carlo simulation was performed using Crystal Ball software.

## 3 Results

### 3.1 Characteristics of HMs in soil

The physicochemical properties and amounts of potentially toxic elements (PTEs) in soil samples are shown in Table 1. Among the eight HMs detected, only Hg (20%), As (48%), Cd (28%), Cu (8%), Ni (4%), and Zn (70%) exceeded the background values in Chongqing, with Zn showing the most severe exceedance (59). According to the Soil Environmental Quality—Risk Control Standard for Soil Contamination of Agricultural Land in China (GB 15618-2018) (60), only 14% of the soil samples for Cd exceeded the risk screening values in the study area, indicating an enrichment of soil Cd in the region (Supplementary Table S2). This enrichment is likely associated with rapid urbanization and industrialization, which causes pollutants to enter the soil through atmospheric deposition, leading to elevated levels (61). The coefficient of variation (CV) is used to assess whether HMs are evenly distributed and can reflect the influence of anthropogenic activities (62). The CVs for Pb, Hg, As, Cr, Cd, Cu, Ni, and Zn were 16.8, 103, 29.8, 13.9, 43.6, 36.0, 16.9, and 34.3%, respectively. The CV for Hg was >1, indicating significant spatial variability in its distribution, suggesting anthropogenic activities are the primary source of this variation.

**Table 1:** Descriptive statistics for soil potentially toxic elements and soil physicochemical properties (*n* = 50; units in mg·kg^−1^).

**PTE**	**Max**	**Min**	**Mean**	**SD**	**CV (%)**	**Skewness**	**Kurtosis**	**Background value**
pH	7.80	4.40	6.13	0.820	13.4			
Pb	27.1	12.6	20.2	3.38	16.8	0.0300	−0.550	28.1
Hg	0.340	0.0200	0.0600	0.0600	103	3.28	11.2	0.0700
As	11.8	2.94	6.78	2.02	29.8	0.490	0.200	6.62
Cr	73.2	26.8	60.0	8.37	13.9	−1.59	4.77	74.4
Cd	0.760	0.0700	0.260	0.110	43.6	1.91	6.58	0.280
Cu	42.0	8.90	16.7	6.01	36.0	1.99	5.77	24.6
Ni	33.4	11.0	25.6	4.33	16.9	−0.960	1.74	31.6
Zn	203	19.8	96.8	33.2	34.3	1.49	2.93	81.9

### 3.2 Spatial distribution characteristics of HMs contamination in soil

**Figure 2:**
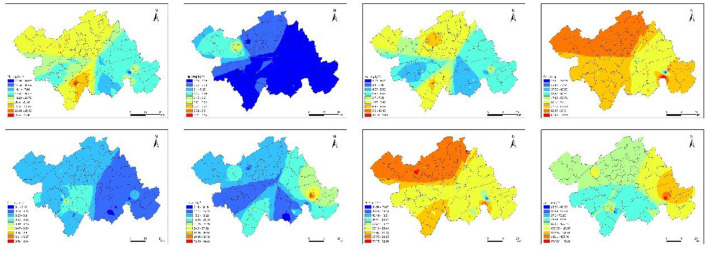
Spatial distribution of eight HM contents in soil of study area.

The spatial distributions of the contents of HMs (Pb, Hg, As, Cr, Cd, Cu, Ni, Zn) in the study area were obtained using the Kriging interpolation method (Figure 2). The spatial distribution of Pb and As showed a clear similarity, mainly concentrated in the northern and southwestern parts of the study area. Sampling points with high Cr and Ni concentrations were mainly distributed in parts of the northwest, southwest, and southeast, with their spatial distribution showing similar patterns. The areas with high Cu and Zn values were both concentrated in the eastern part of the study area, with their spatial distribution showing similar patterns. Areas with high Hg and Cd concentrations were relatively few, occurring only in sporadic spots. The area with a high value for Hg was located in the northwest, while the area with a high value for Cd was in the southwest.

### 3.3 HMs contamination in soil

**Figure 3:**
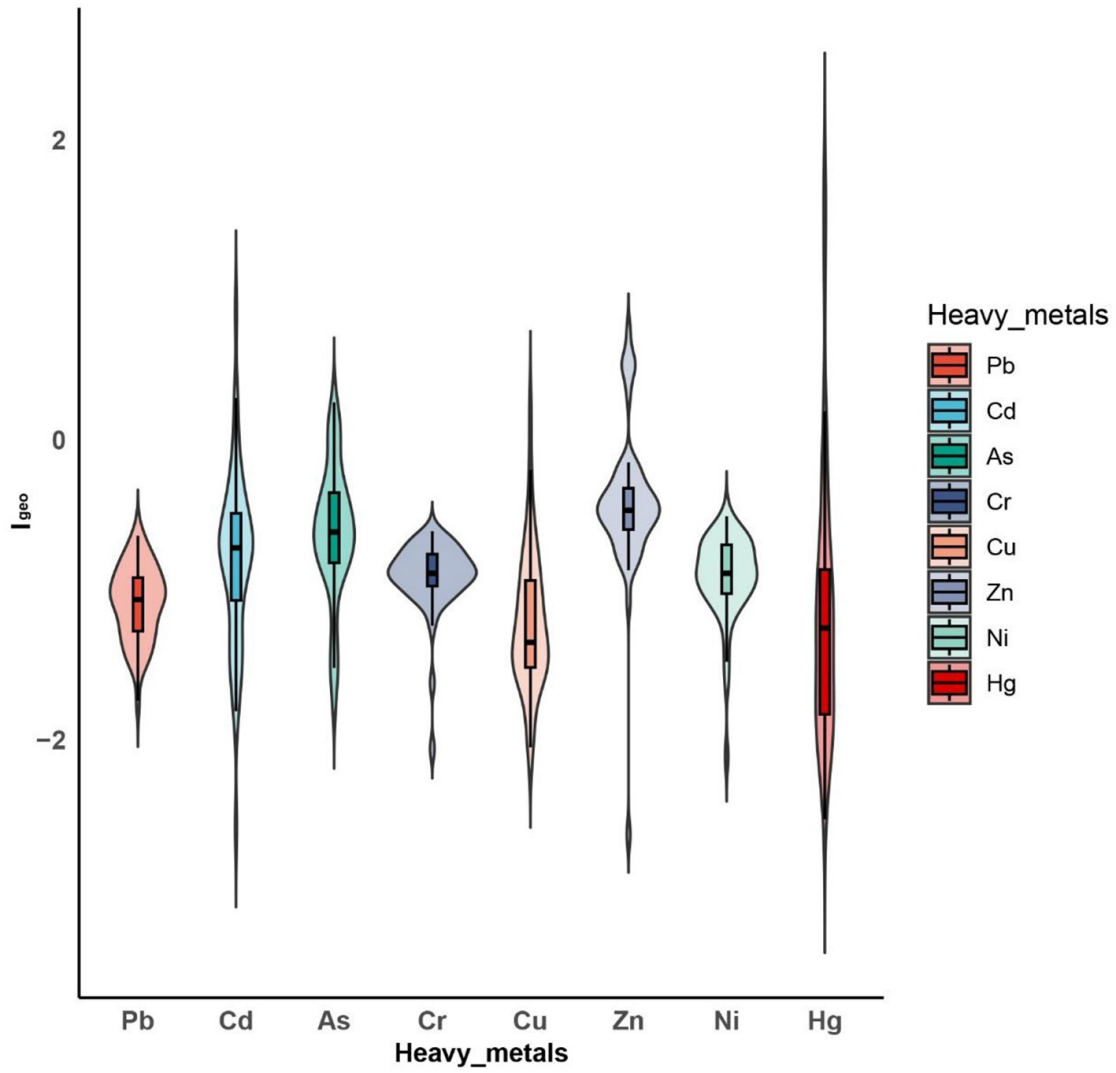
Violin plots of *I*_geo_ values of eight HMs in the study regions.

Figure 3 and Supplementary Table S3 show the *I*_geo_ of eight HMs in 50 soil samples. The mean and standard deviation of the *I*_geo_ of Pb, Hg, As, Cr, Cd, Cu, Ni and Zn were −1.08 ± 0.25, −1.15 ± 0.91, −0.61 ± 0.45, −0.91 ± 0.24, −0.80 ± 0.59, −1.22 ± 0.45, −0.91 ± 0.28, and −0.42 ± 0.50, respectively. The max *I*_geo_ values of Pb, Cr, and Ni were below 0, suggesting that these metals did not contaminate the soil in the study area. As, Cd, Cu, and Zn showed mild contamination in 12, 6, 2, and 12% of the samples, respectively. Mild and moderate Hg contamination was found in 4 and 6%, respectively. These findings indicate that the soil in the study area was mainly contaminated by Hg, As, Cd, Cu, and Zn, with Hg having a higher contamination risk.

### 3.4 Crops HM contents and bioaccumulation factor evaluation

The characteristics of HMs contents in crops are shown in Table 2. The content of Pb in crops was within the safety limits specified by the Chinese National Food Safety Standards for Contaminants in Food (63), and the contents of Cu and Zn in crops were below the thresholds set by the Chinese Health Standards for Cu and Zn limits in food (64, 65). In crops, a small number of samples for Hg, As, and Cd showed standard exceedances, with exceedance rates of 6, 6, and 8%, respectively. However, Cr and Ni exhibited significant exceedances, with more than half of the samples exceeding the standards for these elements, which requires attention. Compared with other crops, the concentrations of As, Cu, Zn, and Hg in grains were significantly higher (*P* < 0.05), which may be related to differences in HM accumulation patterns among crops.

**Table 2:** Statistics of HM contents in crops (mean ± SD, mg·kg^−1^).

**Types**	**Pb**	**Hg**	**As**	**Cr**	**Cd**	**Cu**	**Ni**	**Zn**
Grains	0.0562 ± 0.0186	0.0111 ± 0.0125	0.209 ± 0.162	1.50 ± 0.742	0.0720 ± 0.0833	2.06 ± 0.767	0.919 ± 0.443	16.3 ± 5.76
Vegetables	0.0547 ± 0.0127	0.000300 ± 0.000500	0.0737 ± 0.0948	1.01 ± 1.40	0.0527 ± 0.0713	1.27 ± 0.432	0.658 ± 0.908	8.29 ± 6.61
Leaf vegetables	0.0795 ± 0.0193	0.000300 ± 0.000500	0.0233 ± 0.0404	0.857 ± 1.40	0.0486 ± 0.0609	1.63 ± 0.949	0.517 ± 0.895	7.15 ± 8.97
Tubers	0.0524 ± 0.0109	0.000600 ± 0.00130	0.105 ± 0.157	0.627 ± 0.808	0.0263 ± 0.0183	1.22 ± 0.321	0.444 ± 0.468	6.91 ± 5.71

**Figure 4:**
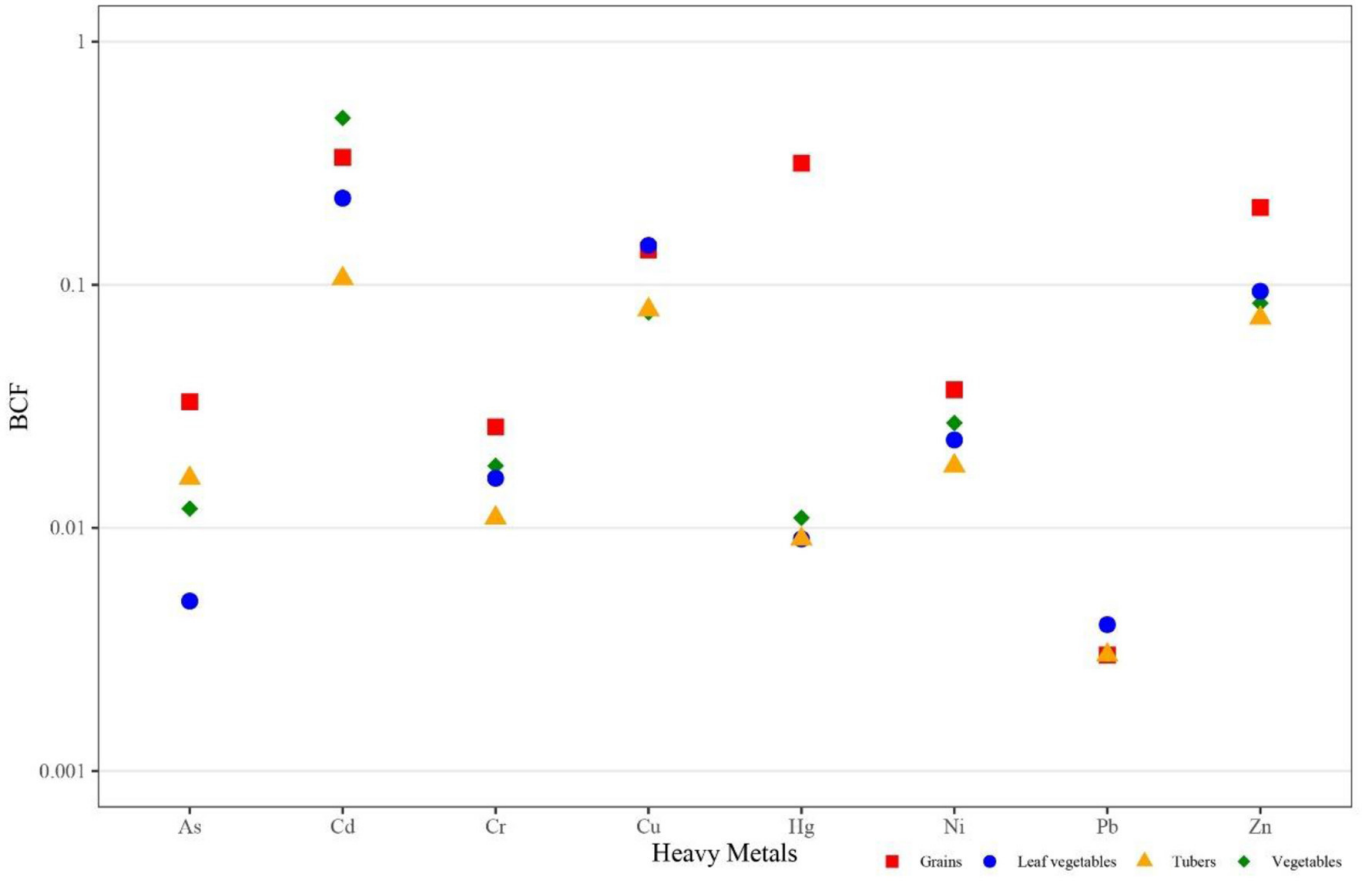
Bioaccumulation factor of eight HMs in different kinds of crops.

The BCF serves as a valuable indicator for assessing the ability of crops to uptake HMs from the soil, providing a quantitative evaluation of their cumulative characteristics and potential harm to crops (66). The study showed significant differences in the enrichment capacity for eight HMs among different crops (Figure 4 and Supplementary Table S4). These differences were largely attributed to the unique growth habits of each crop and their varying tolerance levels to different HMs. Additionally, the absorption of different HMs by crops may involve synergistic or antagonistic effects, further influencing the enrichment capacity of different crops for HMs. Among all crops, grains demonstrated a higher enrichment capacity for eight HMs, which may be related to their longer growth cycles. The enrichment capacity for As, Cu, Zn, and Hg in grains was significantly higher than in other crops (*P* < 0.05). Furthermore, this study found that the average enrichment coefficients for Cd across four types of crops were relatively high, likely due to the higher content of Cd in the local soil (61).

### 3.5 IICQ index evaluation

**Figure 5:**
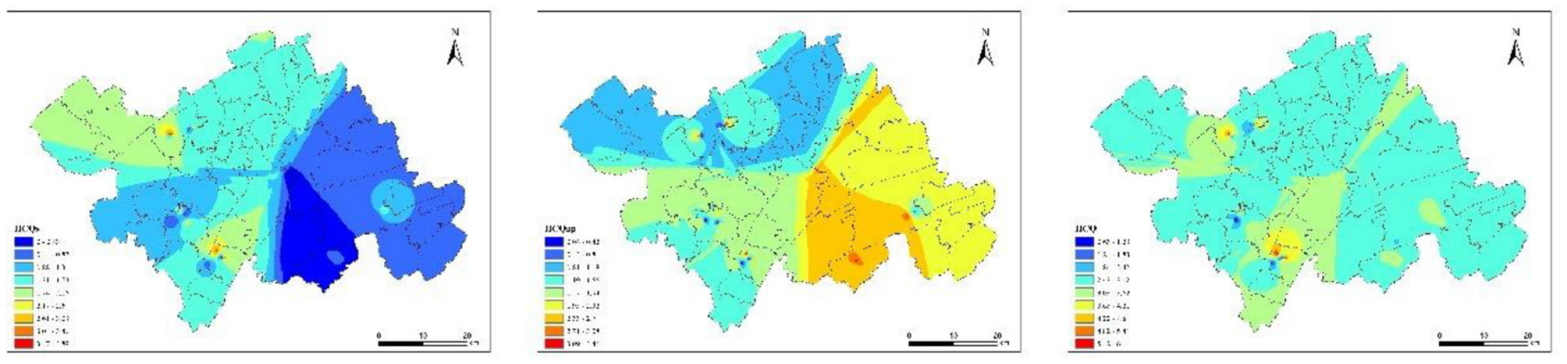
IICQ index spatial distribution.

The average IICQ value for the soil-crop system was 2.97, with pollution levels classified as clean, slight, light, moderate, and heavy in 6, 18, 40, 26, and 10% of the samples, respectively. The mean IICQ_s_ value for soil HMs was 1.15, with no heavy pollution in all samples. The soil was clean, slightly polluted, moderately polluted, and heavily polluted in 54, 30, 6, and 10% of the samples, respectively. For crops, the mean IICQ_ap_ value was 1.82, with no samples showing heavy pollution. The majority of the samples had light pollution (52%), followed by clean (26%), moderate pollution (12%), and slight pollution (10%; Supplementary Table S5). Crops HMs contributed to approximately 61.28% of the overall IICQ for the soil-crop system, explaining crops as the main source of HM exposure. However, soil still contributed 38.72% of HM exposure. The study found differences in the average IICQ_ap_ values among various types of crops. The average IICQ_ap_ value for leaf vegetables was 0.83, indicating no pollution. The average IICQ_ap_ values for vegetables and tubers were 1.38 and 1.20, respectively, indicating slight pollution levels. The average IICQ_ap_ value for grains was 2.36, indicating light pollution levels (Supplementary Table S6). The spatial distribution of the IICQ values (Figure 5) revealed higher IICQ_s_ in the northwest and southwest regions, while higher crop IICQ_ap_ values were mainly observed in the southeast. The spatial correlation between IICQ_s_ and IICQ_ap_ was low, which may be influenced by the types of crops cultivated and their growth habits (67), causing the spatial distribution of IICQ_s_ and IICQ_ap_ to be inconsistent.

### 3.6 Correlation analysis of HMs in soil-crop systems

**Figure 6:**
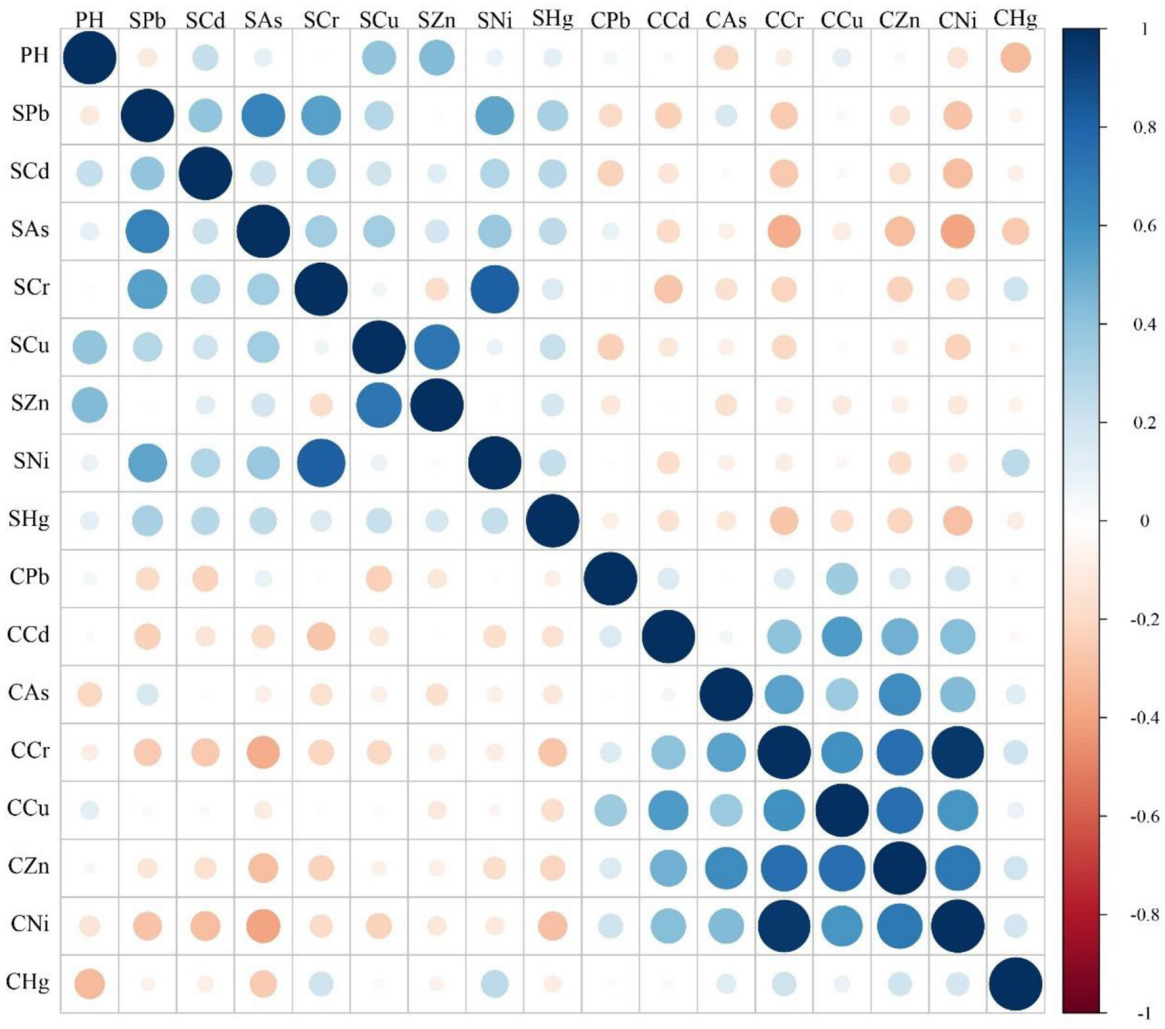
Correlation matrix of eight HMs of soil and crop in soil-crop systems. S represents soil, and C represents crop.

Pearson's correlation analysis results of eight HMs in the soil-crop system are shown in Figure 6 and Supplementary Table S7. HMs in soil and crops were significantly correlated at the 0.01 or 0.05 levels. The correlation analysis results showed that the pH value had a weak positive correlation with Cu in soil (*P* < 0.01), a moderate positive correlation with Zn in soil (*P* < 0.01), and a weak negative correlation with Hg in crops (*P* < 0.05). Pb in the soil had a moderate positive correlation with Cr and Ni in soil (*P* < 0.01), a strong positive correlation with As in soil (*P* < 0.01), and a weak positive correlation with Cd (*P* < 0.01), Cu (*P* < 0.05), and Hg (*P* < 0.05) in soil, and a weak negative correlation with Ni in crops (*P* < 0.05). Cd in the soil had a weak positive correlation with Cr and Ni in soil (*P* < 0.05) and a weak negative correlation with Ni in crops (*P* < 0.05). As in soil had a weak positive correlation with Cr (*P* < 0.05), Cu (*P* < 0.05), and Ni (*P* < 0.01) in soil, and a weak negative correlation with Cr (*P* < 0.01), Ni (*P* < 0.01), and Zn (*P* < 0.05) in crops. Cr in soil had a very strong positive correlation with Ni in soil (*P* < 0.01). Cu in the soil had a strong positive correlation with Zn in soil (*P* < 0.01). Hg in the soil had a weak negative correlation with Ni in crops (*P* < 0.05). Pb in crops had a weak positive correlation with Cu in crops (*P* < 0.05). Cd in crops had a moderate positive correlation with Cr, Cu, Zn, and Ni in crops (*P* < 0.01). As in crops had a moderate positive correlation with Cr and Ni in crops (*P* < 0.01), a weak positive correlation with Cu in crops (*P* < 0.01), and a strong positive correlation with Zn in crops (*P* < 0.01). Cr in crops had a moderate, strong, and extremely strong positive correlation with Cu (*P* < 0.01), Zn (*P* < 0.01), and Ni (*P* < 0.01) in crops, respectively. Cu in crops had a strong and moderate positive correlation with Zn (*P* < 0.01) and Ni (*P* < 0.01) in crops, respectively. Zn in crops had a strong positive correlation with Ni in crops (*P* < 0.01).

**Figure 7:**
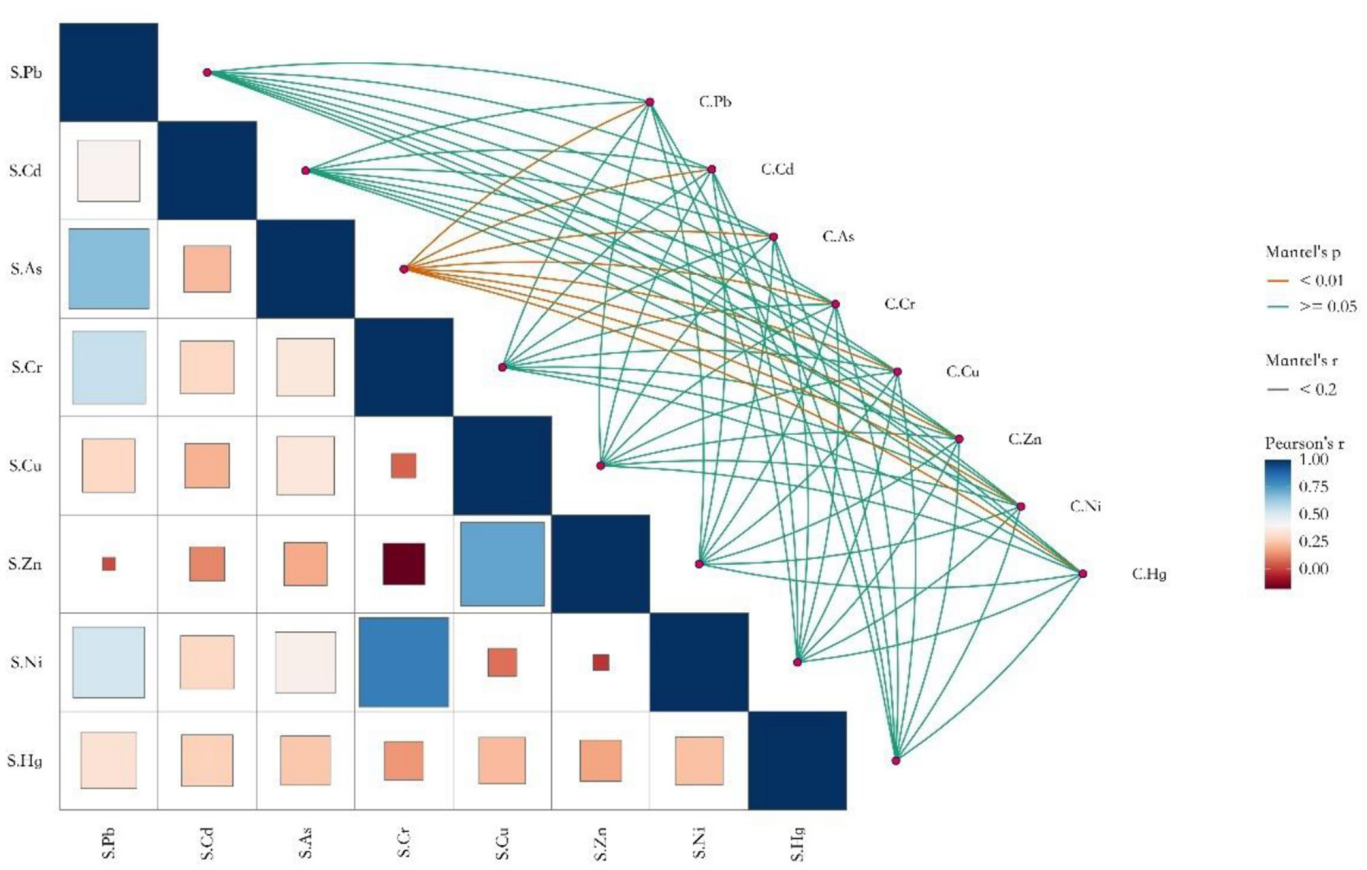
Mantel test of eight HMs of soil and crop in soil-crop systems. S represents soil, and C represents crop.

The results of the Mantel test for the eight HMs in the soil-crop system are shown in Figure 7. The right half of the graph shows the results of the Mantel test analysis, displaying the correlation between the concentrations of HMs in soil and crops. The left half of the graph shows the correlation of the concentrations of HMs in soil. The figure displays the correlations among different factors within the same matrix and between different matrices. There was a significant correlation (*P* < 0.01) between As in soil and eight HMs in crops, but the strength of the correlation was weak (*r* < 0.2). Other HMs in the soil did not show significant correlations with HMs in crops (*P* > 0.05, *r* < 0.2).

### 3.7 Health risk assessment of HMs in crops based on Monte Carlo model

#### 3.7.1 Uncertainty analysis of non-carcinogenic health risk

**Figure 8:**
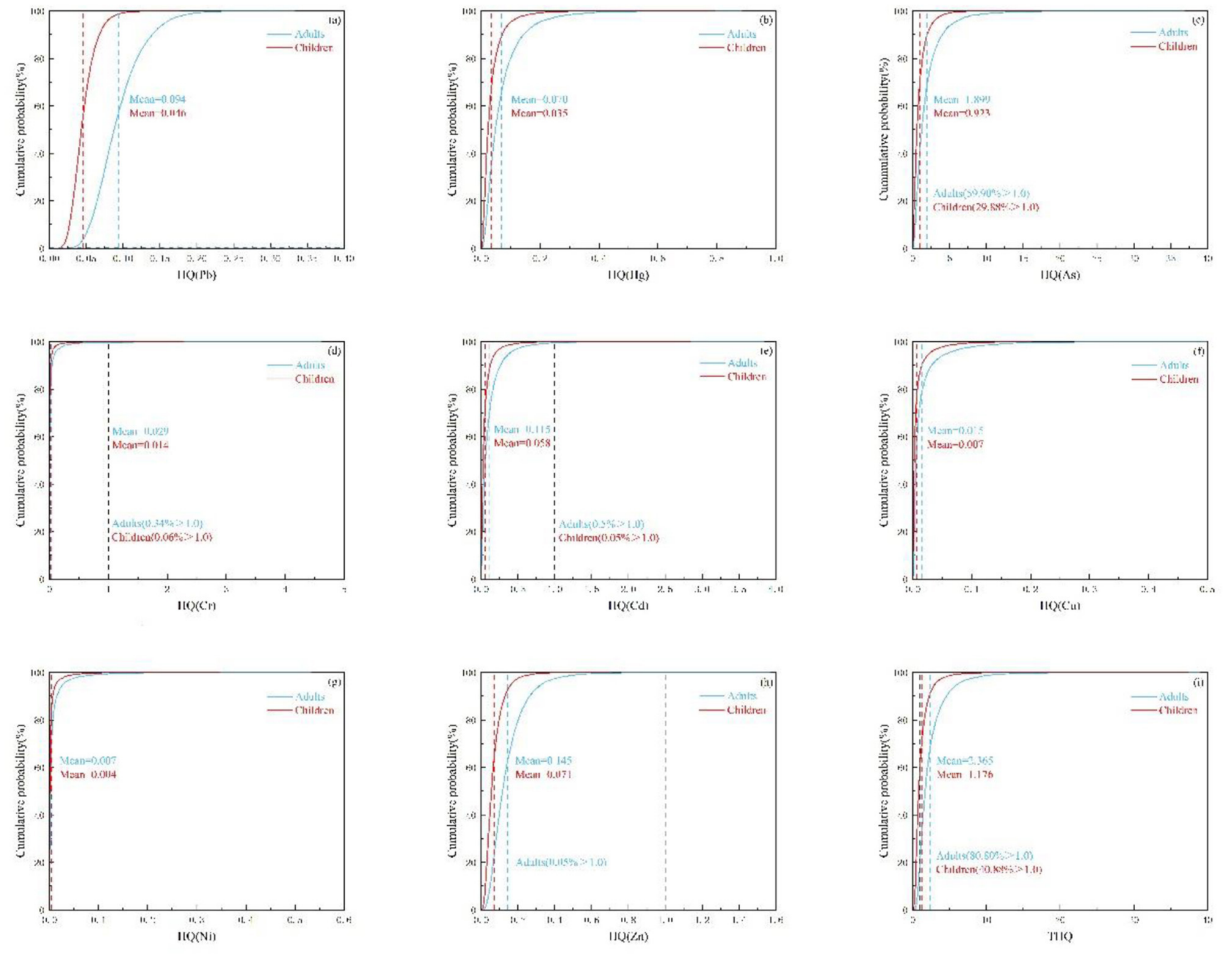
The probability distribution for health risk index of non-carcinogenic HMs in crops. Hazard quotient (HQ) for **(a)** Pb, **(b)** Hg, **(c)** As, **(d)** Cr, **(e)** Cd, **(f)** Cu, **(g)** Ni, **(h)** Zn, and index for **(i)** total hazard quotient (THQ) [the red and blue dashed lines indicated the mean values, while the black indicated the guideline value for non-carcinogenic risk (1.0)].

For non-carcinogenic risks, the rank of the average HQ values was As > Zn > Cd > Pb > Hg > Cr > Cu > Ni for both children and adults (Figures 8a–h). Among these HMs, the average HQ values of As were 1.899 and 0.923 for adults and children, with about 59.9 and 29.88% of crops samples exceeding 1.0, respectively (Figure 8c). The average HQ values for other HMs were lower than 1.0, with only a few HQ values for Cd and Cr exceeding 1.0. Besides, the average THQ value for adults was 2.365 [90% confidence interval (CI): 0.656–6.104], and the average THQ value for children was 1.176 (90% CI: 0.324–3.028; Supplementary Table S8), which were far higher than the guideline threshold (1.0). The excess rates were 80.80% for adults and 40.88% for children (Figure 8i).

#### 3.7.2 Uncertainty analysis of carcinogenic health risk

**Figure 9:**
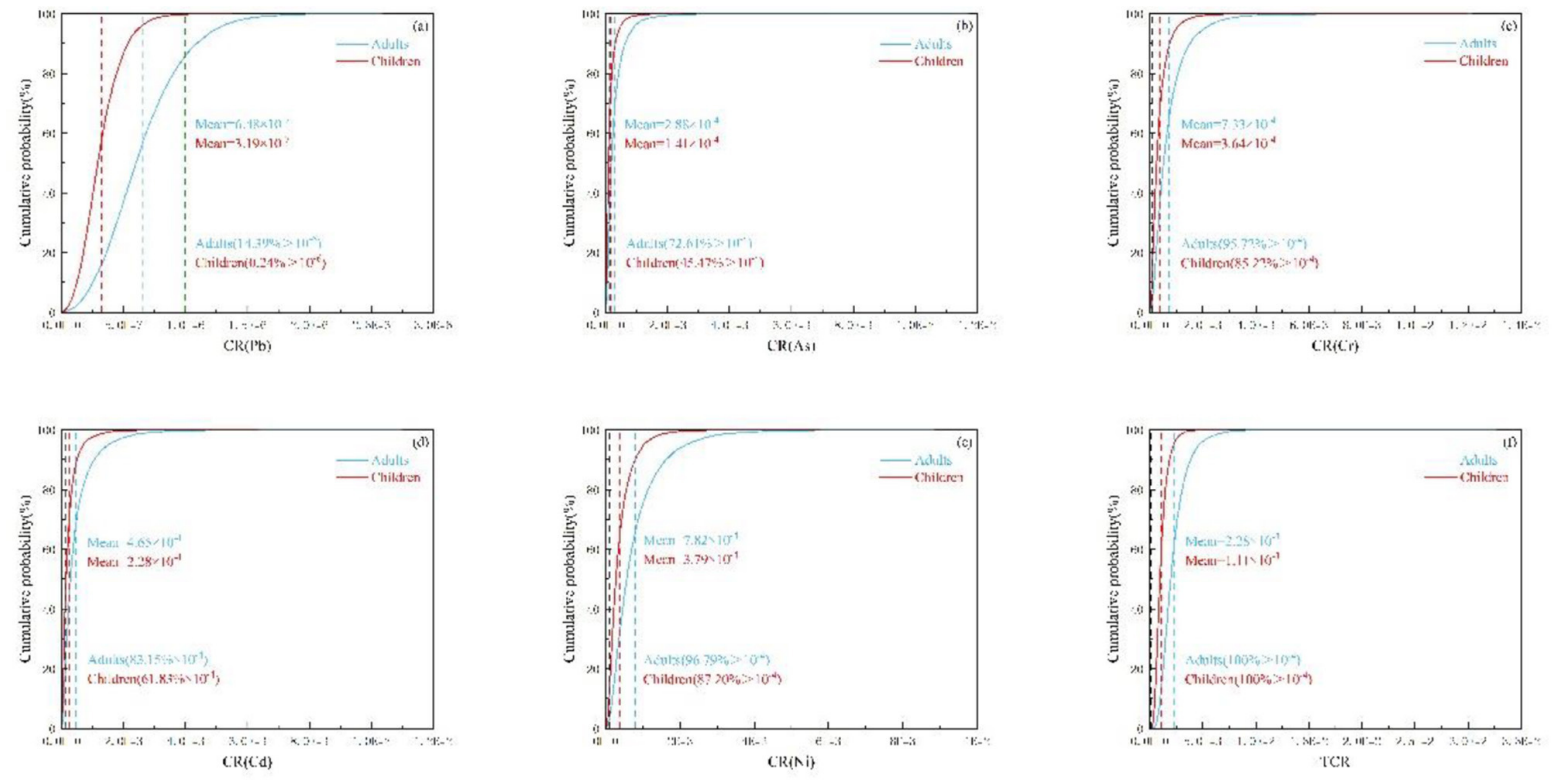
The probability distribution for health risk index of carcinogenic HMs in crops. Carcinogenic risk (CR) for **(a–e)**, and index for **(f)** total carcinogenic risk (TCR) [the red and blue dashed lines indicated the mean values, while the black indicated the guideline value for carcinogenic risk (10^−4^). The green dashed lines presented the acceptable carcinogenic risks value that of 10^−6^].

For carcinogenic risks, the average CR values followed the order of Ni > Cr > Cd > As > Pb for both adults and children (Figures 9a–f). The average CR values of the above HMs (except Pb) all exceeded the unacceptable risk of 1.0 × 10^−4^. However, only about 14.39% (adults) and 0.24% (children) of CR values for Pb were acceptable (Figure 9a). Additionally, the average TCR value for adults was 2.28 × 10^−3^ (90% CI: 8.33 × 10^−4^ to 4.65 × 10^−3^), and the average TCR value for children was 1.11 × 10^−3^ (90% CI: 4.06 × 10^−4^ to 2.27 × 10^−3^). Nearly 100% of the TCR values surpassed 1.0 × 10^−4^ (Supplementary Table S8).

#### 3.7.3 Sensitivity analysis of Monte Carlo model

**Figure 10:**
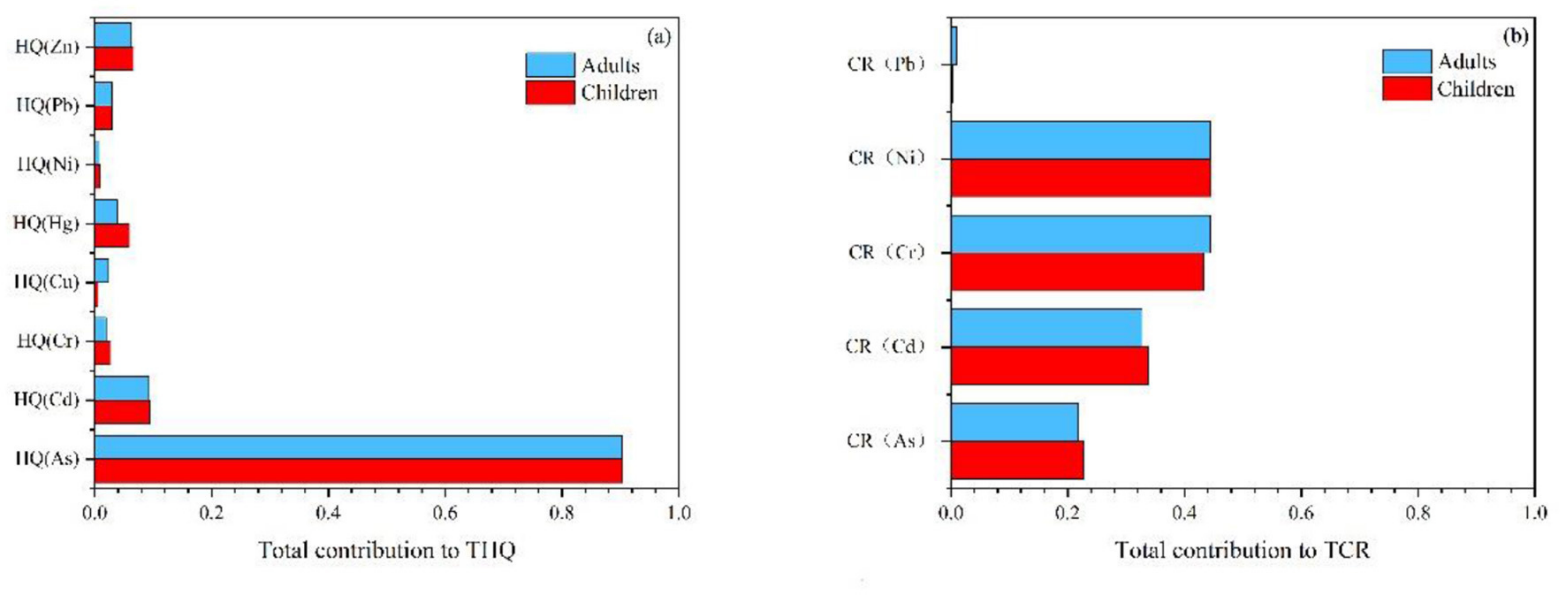
Sensitivity analyses of HMs for **(a)** total hazard quotient and **(b)** total carcinogenic risk.

Sensitivity analysis was conducted to estimate the weight of each parameter in health risk assessment. The larger the sensitivity value, the greater the impact on risk. The results indicated that the concentration (C) of HMs in crops was a crucial exposure parameter influencing the health risks for adults and children. For the HQ value, the exposure frequency also had an impact, contributing 18.0% to the variance of HQ in adults and 17.7% in children. Parameters such as body weight and oral reference dose (RfD) were negatively correlated with health risk. The carcinogenic risk was also influenced by the cancer slope factor (SF) and exposure frequency (EF). The slope factor contributed to 48.8 and 49.6% of the variance in CR for adults and children, respectively, while exposure frequency contributed to 17.2% for adults and 17.1% for children. Body weight contributed < 10% to health risk (Supplementary Tables S9, S10). Among the analyzed HMs, As contributed the most to the total non-carcinogenic risk, with 90.29% for adults and 90.40% for children. In terms of TCR, Ni, Cr, and Cd contributed more than As, with Ni at 44.40% for adults and 44.53% for children, Cr at 44.43% for adults and 43.26% for children, and Cd at 32.79% for adults and 33.88% for children (Figure 10). Overall, As, Ni, Cr, and Cd contributed significantly to the total health risk.

## 4 Discussion

### 4.1 Factors influencing the distribution of heavy metal concentrations in crops and soil

In our study, the concentration of Zn in soil samples was higher than the background values in Chongqing in many cases and the average concentration of Zn in a study on HM pollution in Chinese agricultural and urban soils over the past 20 years; however, it was lower than the concentration of Zn in the HM risk assessment of the soil-crop system along the Yangtze River in Nanjing (53, 68). Greater attention is required for the enrichment of Cd in soil. This enrichment is related to the widespread occurrence of carbonate rocks rich in Cd in the southwestern provinces of China and the impact of urbanization, which leads to the irrigation of sewage and excessive use of chemical fertilizers and pesticides. Unlike the concentrations of HM in the soil, the exceedance of Cr and Ni in crops is more severe. In this study, the content of Cr in crops was higher than that in crops in Gansu, China, and lower than that in crops near non-ferrous metal mining areas in Yunnan (69, 70). The content of Ni in crops was higher than that in grains in Anhui Province, China, and lower than that in vegetables planted in roadside soils in Pakistan (71, 72). This finding indicates that the concentration of HMs in crops is related to the type and growth characteristics of the crops and correlates with the pollution levels of HMs in soil (73–76). Further analysis revealed a significant link between soil arsenic (As) and its accumulation in graminous crops, particularly rice. This may relate to the structure of the iron plaque on rice roots. In As-contaminated soils, this plaque might enhance arsenic uptake (77). Under flooded conditions, soil As (V) is easily reduced to As (III), which has weaker adsorption to soil minerals than As (V). This leads to higher soil As concentrations and easier uptake by rice (78).

### 4.2 Health risks for different populations through crop ingestion

HMs in crops can be transferred to the human body through the food chain, leading to several health risks. The accumulation of various HMs in crops is a relatively complex process influenced by external environmental and anthropogenic factors. HMs enter crops through contaminated agricultural soil, sewage, atmospheric deposition, and fertilizers containing HMs (79, 80). Through the absorption mechanism of crop root systems, HMs are transferred to various parts of the plant, such as tubers, leaves, fruits, and seeds, by specific transport proteins (81). Long-term consumption of crops contaminated with HMs poses health risks to human health, including kidney damage, osteoporosis, neurological damage, cardiovascular diseases, and increased cancer risk (82–86). The unique physiological characteristics of children predispose them to the toxic effects of HMs. Long-term intake of crops contaminated with HMs can have a negative impact on their cognitive development and physical growth (87, 88). In this study, the average THQ for adults and children were 2.365 and 1.176, respectively, which were greater than the standard value of 1.0, suggesting that consuming crops is associated with a higher non-carcinogenic risk. The average THQ in this study was much higher than the values of 0.0441 for adults and 0.324 for children near the coal mines in Jinzhong City, Shanxi Province (89). Compared to the THQ values in this study, a study on HM contamination in crops in Iran reported a lower value for adults (1.67) but a slightly higher value for children (1.28) (90). Only As had an average HQ value exceeding the standard value of 1.0 in 59.90% of samples for adults, while As had an average HQ value close to the standard value of 1.0 in 29.88% of samples for children. This finding indicates that As poses a significant health risk to adults, but the health risk to children should not be ignored. Cd (adults: 0.5%, children: 0.05%), Cr (adults: 0.34%, children: 0.06%), Zn (children: 0.05%) had average HQ values below the standard value of 1.0, suggesting that Cd, Cr, and Zn may not pose a health risk to the population at most exposure levels, but the cumulative effect still warrants attention (91, 92).

The average TCR in this study was 2.28 × 10^−3^ for adults and 1.11 × 10^−3^ for children, both significantly higher than the unacceptable level of 1.0 × 10^−4^, indicating that HM pollution in crops is prone to carcinogenic risk. The TCR values for both adults and children were lower than the values of 2.92 × 10^−3^ for adults and 4.96 × 10^−3^ for children near the Leng-shuikeng mine in Jiangxi (93). However, the TCR for children in this study was higher than that reported in agricultural soil in Lanzhou (94). For individual HMs, As (adults: 72.61%, children: 45.47%), Cr (adults: 95.72%, children: 85.22%), Cd (adults: 83.15%, children: 61.83%), and Ni (adults: 96.79%, children: 87.20%) had average CR values greater than the standard value of 1.0 × 10^−4^, indicating that these metals pose a higher carcinogenic risk to the population and require attention (25, 95–97).

Several studies show that adults are at greater health risk than children due to higher crop consumption and longer exposure time (98–100). However, other studies demonstrate that children are at greater health risk than adults due to their lower body weight and poorer tolerance to toxic HMs (101–103). HMs affect public health through various routes, such as crops, drinking water, soil, and air pollution (104). Therefore, efforts to identify and control the key exposure factors in crops will reduce the population health risks.

### 4.3 The impact sensitivity analysis using Monte Carlo simulation

Health risk assessments for HM hazards based on Monte Carlo simulation involved many uncertainties. Differences in regions, ethnicities, and ages often lead to variations in model parameters (e.g., exposure frequency and body weight), which can cause deviations in actual health risk levels (105–107). Identifying exposure parameters that significantly impact health risk assessments is essential for reducing uncertainties in the risk assessment process. Reporting the influence and sensitivity of the assessment results can lead to a more accurate evaluation of the impact of heavy metals on population health risks. This study focused on the differences in health risks between adults and children and selected parameters related to human exposure for Monte Carlo simulation and sensitivity analysis. The findings showed no significant differences between the parameters. For both non-carcinogenic and carcinogenic risk assessments, crop intake was found to be the most sensitive parameter. This finding is consistent with previous studies on rice HM pollution in the Minxi region and vegetable HM pollution in Chongqing (56, 61). Sensitivity analysis can help identify key exposure parameters, and when combined with Monte Carlo simulation, it can effectively assess the health risks of HM pollution in different population groups.

## 5 Conclusions

This study provides a systematic evaluation of HM pollution in the fragmented farmland soil-crop system in Wanzhou District, Chongqing City. The results show that grains have a greater capacity to accumulate HMs compared to other crops. In the soil-crop system, over 70% of the samples had IICQ values indicating light pollution levels or higher. Both children and adults are exposed to high levels of non-carcinogenic and carcinogenic risks. As was the chief contributor to non-carcinogenic risk, while Ni, Cr, Cd, and As significantly contributed to the carcinogenic risk. Considering the concentration of HMs, pollution risks, and health hazards, As, Ni, Cr, and Cd should be listed for enhanced monitoring. Finally, we recommend strengthening the monitoring of HMs in crops, adopting proactive and effective regulatory strategies, and reasonably adjusting the dietary intake of residents. These measures are crucial for reducing the hazard of HMs to public health.

## Data Availability

The raw data supporting the conclusions of this article will be made available by the authors, without undue reservation.
