# Supplementary material for: Assessment of heavy metal sources and health risks in soil-crop systems of fragmented farmland
Source: Front Public Health. 2025 Jul 31;13:1637595. doi: 10.3389/fpubh.2025.1637595 (PMC12350282; doi:10.3389/fpubh.2025.1637595)
Supplement: Supplementary file 1 [file Table_1.docx]

Table S1 Parameter values and probability distribution of human health

| Parameter | Description | Unit | Probabilistic distribution | Parameter | Reference |
| --- | --- | --- | --- | --- | --- |
| C_api_ | concentration | mg/kg | Lognormal(Pb) | LN(0.06±0.02) | This study |
|  |  |  | Lognormal(As) | LN(0.15±0.16) |  |
|  |  |  | Lognormal(Cd) | LN(0.06±0.07) |  |
|  |  |  | Lognormal(Cr) | LN(1.16±0.98) |  |
|  |  |  | Lognormal(Hg) | LN(0.01±0.01) |  |
|  |  |  | Lognormal(Ni) | LN(0.73±0.60) |  |
|  |  |  | Lognormal(Cu) | LN(1.69±0.74) |  |
|  |  |  | Lognormal(Zn) | LN(12.04±7.39) |  |
| RfD | oral reference dose | mg/(kg·day) | Triangular(Pb) | TRI (0.0035,0.0035,0.0037） | USEPA, 2011 WHO, 1997 |
|  |  |  | Triangular(As) | TRI (0.0003,0.0003,0.0008） |  |
|  |  |  | Triangular(Cd) | TRI (0.001,0.001,0.01） |  |
|  |  |  | Triangular(Cr) | TRI (0.003,0.003,2.5） |  |
|  |  |  | Triangular(Hg) | TRI (0.0007,0.0007,0.001） |  |
|  |  |  | Triangular(Ni) | TRI (0.02,0.02,5） |  |
|  |  |  | Triangular(Cu) | TRI (0.04,0.04,5） |  |
|  |  |  | Triangular(Zn) | TRI (0.3,0.3,0.91） |  |
| SF | Cancer slope factor | (Kg·day)/mg | Triangular(Cd) | TRI（0,6.1,6.1） | USEPA, 2006 WHO, 1990 |
|  |  |  | Triangular(As) | TRI（0,1.5,1.5） |  |
|  |  |  | Triangular(Pb) | TRI（0,0.0085,0.0085） |  |
|  |  |  | Triangular(Cr) | TRI（0,0.5,0.5） |  |
|  |  |  | Triangular(Ni) | TRI（0,0.84,0.84） |  |
| IR | ingestion rate | kg/day | Point (children) | 0.234 | Zhao,2022 |
|  |  |  | Point (adults) | 0.42 |  |
| BW | body weight | kg | Normal(children) | Norm(17.3±0.21) | Zhao,2022 |
|  |  |  | Normal(adults) | Norm(61.0±0.1) |  |
| EF | exposure frequency | day/year | Triangular | TRI (180, 345, 365) | USEPA, 2011 |
| ED | exposure duration | year | Point (children) | 6 | USEPA, 2011 |
|  |  |  | Point (adults) | 24 |  |
| AT | averaging exposure time | day | Point | 365*ED（non-carcinogenic） | USEPA, 2011 |
|  |  |  |  | 365*70（carcinogenic） |  |

Lognormal distribution, LN (mean, standard deviation); Triangular distribution, TRI (minimum, likeliest, maximum); Normal distribution, Norm (mean, standard deviation).

**References for Table S1**

USEPA, 2011. Exposure Factors Handbook 2011 Edition (Final Report). U.S. Environmental Protection Agency, Washington, DC, EPA/600/R-09/052F.

WHO, 1997. Food Consumption and Exposure Assessment of Chemicals. Report of a FAO/WHO Consultation. World Health Organization, Geneva, Switzerland. Available at: https://apps.who.int/iris/.

USEPA, 2006. Integrated Risk Information System (IRIS). Assessments. U.S. Environmental Protection Agency, Washington, DC, USA. Available at: https://cfpub.epa.gov/ncea/iris_drafts/AtoZ.cfm.

WHO, 1990. IARC Monographs on the Evaluation of Carcinogenic Risks to Humans, vol. 49. Chromium, Nickel and Welding World Health Organization, Lyon. Available at: https://publications.iarc.fr/67.

Zhao, L.Y., Ding, G.Q., Zhao, W.H., 2022. 2015-2017 China Resident Nutrition and Health Status Monitoring Report. People’s Medical Publishing House, Beijing, China.

Table S2 the Soil Environmental Quality—Risk Control Standard for Soil Contamination of Agricultural Land in China

| PTE | Screening value of paddy field | | | | Screening value of others | | | |
| --- | --- | --- | --- | --- | --- | --- | --- | --- |
|  | pH≤5.5 | 5.5<pH≤6.5 | 6.5<pH≤7.5 | pH>7.5 | pH≤5.5 | 5.5<pH≤6.5 | 6.5<pH≤7.5 | pH>7.5 |
| Pb | 80 | 100 | 140 | 240 | 70 | 90 | 120 | 170 |
| Hg | 0.5 | 0.5 | 0.6 | 1 | 1.3 | 1.8 | 2.4 | 3.4 |
| As | 30 | 30 | 25 | 20 | 40 | 40 | 30 | 25 |
| Cr | 250 | 250 | 300 | 350 | 150 | 150 | 200 | 250 |
| Cd | 0.3 | 0.4 | 0.6 | 0.8 | 0.3 | 0.3 | 0.3 | 0.6 |
| Cu | 50 | 50 | 100 | 100 | 50 | 50 | 100 | 100 |
| Ni | 60 | 70 | 100 | 190 | 60 | 70 | 100 | 190 |
| Zn | 200 | 200 | 250 | 300 | 200 | 200 | 250 | 300 |

Table S3 I_geo_ values of heavy metals for the soil samples from Wanzhou District, Chongqing

| PTE | Max | Min | Mean | SD |
| --- | --- | --- | --- | --- |
| Pb | -0.64 | -1.74 | -1.08 | 0.25 |
| Hg | 1.69 | -2.53 | -1.15 | 0.91 |
| As | 0.25 | -1.76 | -0.61 | 0.45 |
| Cr | -0.61 | -2.06 | -0.91 | 0.24 |
| Cd | 0.86 | -2.58 | -0.80 | 0.59 |
| Cu | 0.19 | -2.05 | -1.22 | 0.45 |
| Ni | -0.51 | -2.11 | -0.91 | 0.28 |
| Zn | 0.72 | -2.63 | -0.42 | 0.50 |

Table S4 BCF values of HMs for the soil samples from Wanzhou District, Chongqing

| Types | Pb | Hg | As | Cr | Cd | Cu | Ni | Zn |
| --- | --- | --- | --- | --- | --- | --- | --- | --- |
| Grains | 0.003±0.001 | 0.316±0.463 | 0.033±0.022 | 0.026±0.014 | 0.334±0.457 | 0.139±0.068 | 0.037±0.019 | 0.208±0.149 |
| Vegetables | 0.003±0.001 | 0.011±0.017 | 0.012±0.015 | 0.018±0.026 | 0.485±1.097 | 0.077±0.044 | 0.027±0.037 | 0.084±0.072 |
| Leaf vegetables | 0.004±0.001 | 0.009±0.015 | 0.005±0.009 | 0.016±0.027 | 0.227±0.314 | 0.145±0.117 | 0.023±0.040 | 0.094±0.128 |
| Tubers | 0.003±0.001 | 0.009±0.026 | 0.016±0.025 | 0.011±0.014 | 0.106±0.070 | 0.079±0.029 | 0.018±0.019 | 0.073±0.068 |

Table S5 IICQ Index evaluation results

| Parameter | IICQ_s_ | IICQ_ap_ | IICQ |
| --- | --- | --- | --- |
| Max | 3.90 | 3.92 | 6.34 |
| Min | 0.00 | 0.02 | 0.52 |
| Mean | 1.15 | 1.82 | 2.97 |
| SD | 1.09 | 1.23 | 1.44 |
| clean（%） | 54.00 | 26.00 | 6.00 |
| slight pollution（%） | 30.00 | 10.00 | 18.00 |
| light pollution（%） | 6.00 | 52.00 | 40.00 |
| moderate pollution（%） | 10.00 | 12.00 | 26.00 |
| heavy pollution（%） | 0.00 | 0.00 | 10.00 |

Table S6 Statistics of IICQap for different types of crops

| Types | Max | Min | Mean | SD |
| --- | --- | --- | --- | --- |
| Grains | 3.74 | 0.02 | 2.36 | 0.94 |
| Vegetables | 3.92 | 0.03 | 1.38 | 1.46 |
| Leaf vegetables | 2.45 | 0.02 | 0.83 | 1.40 |
| Tubers | 2.48 | 0.03 | 1.20 | 1.12 |

Table S7 Correlation matrix of heavy metals of soil and crop in soil-crop systems (S represent soil, and C represents crop)

|  | PH | S.Pb | S.Cd | S.As | S.Cr | S.Cu | S.Zn | S.Ni | S.Hg | C.Pb | C.Cd | C.As | C.Cr | C.Cu | C.Zn | C.Ni | C.Hg |
| --- | --- | --- | --- | --- | --- | --- | --- | --- | --- | --- | --- | --- | --- | --- | --- | --- | --- |
| PH | 1 |  |  |  |  |  |  |  |  |  |  |  |  |  |  |  |  |
| S.Pb | -0.119 | 1 |  |  |  |  |  |  |  |  |  |  |  |  |  |  |  |
| S.Cd | 0.232 | .390^**^ | 1 |  |  |  |  |  |  |  |  |  |  |  |  |  |  |
| S.As | 0.108 | .663^**^ | 0.219 | 1 |  |  |  |  |  |  |  |  |  |  |  |  |  |
| S.Cr | -0.014 | .548^**^ | .290^*^ | .346^*^ | 1 |  |  |  |  |  |  |  |  |  |  |  |  |
| S.Cu | .396^**^ | .289^*^ | 0.204 | .345^*^ | 0.060 | 1 |  |  |  |  |  |  |  |  |  |  |  |
| S.Zn | .436^**^ | 0.018 | 0.122 | 0.188 | -0.179 | .722^**^ | 1 |  |  |  |  |  |  |  |  |  |  |
| S.Ni | 0.088 | .521^**^ | .290^*^ | .378^**^ | .818^**^ | 0.083 | -0.026 | 1 |  |  |  |  |  |  |  |  |  |
| S.Hg | 0.110 | .324^*^ | 0.272 | 0.251 | 0.153 | 0.222 | 0.176 | 0.235 | 1 |  |  |  |  |  |  |  |  |
| C.Pb | 0.055 | -0.182 | -0.229 | 0.095 | 0.018 | -0.232 | -0.126 | -0.013 | -0.084 | 1 |  |  |  |  |  |  |  |
| C.Cd | 0.031 | -0.230 | -0.136 | -0.187 | -0.272 | -0.124 | -0.018 | -0.176 | -0.153 | 0.158 | 1 |  |  |  |  |  |  |
| C.As | -0.201 | 0.161 | -0.023 | -0.084 | -0.162 | -0.082 | -0.160 | -0.080 | -0.126 | 0.017 | 0.054 | 1 |  |  |  |  |  |
| C.Cr | -0.090 | -0.252 | -0.269 | -.370^**^ | -0.218 | -0.203 | -0.095 | -0.097 | -0.274 | 0.145 | .401^**^ | .530^**^ | 1 |  |  |  |  |
| C.Cu | 0.112 | -0.034 | 0.031 | -0.107 | -0.019 | -0.027 | -0.114 | -0.042 | -0.172 | .352^*^ | .562^**^ | .365^**^ | .600^**^ | 1 |  |  |  |
| C.Zn | 0.041 | -0.135 | -0.160 | -.301^*^ | -0.222 | -0.071 | -0.087 | -0.173 | -0.214 | 0.153 | .473^**^ | .624^**^ | .757^**^ | .758^**^ | 1 |  |  |
| C.Ni | -0.143 | -.281^*^ | -.304^*^ | -.399^**^ | -0.180 | -0.225 | -0.124 | -0.116 | -.291^*^ | 0.208 | .428^**^ | .436^**^ | .969^**^ | .584^**^ | .720^**^ | 1 |  |
| C.Hg | -.312^*^ | -0.061 | -0.082 | -0.251 | 0.201 | -0.036 | -0.065 | 0.260 | -0.097 | -0.020 | -0.033 | 0.130 | 0.202 | 0.090 | 0.194 | 0.182 | 1 |
| *. At the 0.05 level (two-tailed), the correlation is significant. | | | | | | | | | | | | | | | | | |
| **. At the 0.01 level (two-tailed), the correlation is significant. | | | | | | | | | | | | | | | | | |

Table S8 Summary statistics for health metals of health risk results via Monte Carlo simulation

| Metal | Hazard quotient (HQ) | | Carcinogenic risk (CR) | |
| --- | --- | --- | --- | --- |
|  | Adults | Children | Adults | Children |
| Pb | 0.094(0.048,0.158) | 0.046(0.024,0.077) | 6.48×10-7(1.84×10-7,1.26×10-6) | 3.19×10-7(8.86×10-8,6.26×10-7) |
| Hg | 0.070(0.012,0.196) | 0.035(0.006,0.098) |  |  |
| As | 1.899(0.269,5.644) | 0.923(0.137,2.763) | 2.88×10^-4^(3.20×10^-5^,8.74×10^-4^) | 1.41×10^-4^(1.51×10^-5^,4.46×10^-4^) |
| Cr | 0.029(0.001,0.095) | 0.014(0.001,0.047) | 7.33×10^-4^(1.10×10^-4^,2.06×10^-3^) | 3.64×10^-4^(5.23×10^-5^,1.01×10^-3^) |
| Cd | 0.115(0.010,0.386) | 0.058(0.005,0.196) | 4.65×10^-4^(4.61×10^-5^,1.52×10^-3^) | 2.28×10^-4^(2.23×10^-5^,6.98×10^-4^) |
| Cu | 0.015(0.002,0.058) | 0.007(0.001,0.029) |  |  |
| Ni | 0.007(0.000,0.028) | 0.004(0.000,0.014) | 7.82×10^-4^(1.25×10^-4^,2.16×10^-3^) | 3.79×10^-4^(5.97×10^-5^,1.02×10^-3^) |
| Zn | 0.145(0.040,0.331) | 0.071(0.020,0.166) |  |  |
| Total | 2.365(0.656,6.104) | 1.176(0.324,3.028) | 2.28×10^-3^(8.33×10^-4^,4.65×10^-3^) | 1.11×10^-3^(4.06×10^-4^,2.27×10^-3^) |
| Meaning of the values: mean (90% confidence interval) | | | | |

Table S9 Sensitivity analyses of health risk parameters for hazard quotient (HQ)

| Metal | Concentration (C) | | Oral reference dose (RfD) | | Body weight (BW) | | Exposure frequency(EF) | |
| --- | --- | --- | --- | --- | --- | --- | --- | --- |
|  | Adults | Children | Adults | Children | Adults | Children | Adults | Children |
| Pb | 0.900 | 0.896 | -0.017 | -0.043 | 0.001 | -0.033 | 0.396 | 0.399 |
| Hg | 0.978 | 0.977 | -0.107 | -0.106 | -0.004 | -0.014 | 0.165 | 0.173 |
| As | 0.945 | 0.945 | -0.274 | -0.267 | 0.002 | -0.006 | 0.167 | 0.149 |
| Cr | 0.580 | 0.588 | -0.755 | -0.756 | 0.004 | -0.018 | 0.116 | 0.101 |
| Cd | 0.832 | 0.824 | -0.514 | -0.530 | -0.011 | -0.008 | 0.121 | 0.133 |
| Cu | 0.413 | 0.403 | -0.877 | -0.874 | -0.010 | -0.011 | 0.145 | 0.140 |
| Ni | 0.586 | 0.591 | -0.764 | -0.765 | 0.003 | -0.013 | 0.109 | 0.121 |
| Zn | 0.860 | 0.857 | -0.421 | -0.434 | 0.008 | -0.036 | 0.223 | 0.201 |
| Mean | 0.762 | 0.760 | -0.466 | -0.472 | -0.001 | -0.017 | 0.180 | 0.177 |

Table S10 Sensitivity analyses of health risk parameters for Carcinogenic risk (CR)

| Metal | Concentration (C) | | Cancer slope factor (SF) | | Body weight (BW) | | Exposure frequency(EF) | |
| --- | --- | --- | --- | --- | --- | --- | --- | --- |
|  | Adults | Children | Adults | Children | Adults | Children | Adults | Children |
| Pb | 0.581 | 0.575 | 0.702 | 0.708 | 0.013 | -0.004 | 0.270 | 0.261 |
| As | 0.862 | 0.864 | 0.411 | 0.430 | -0.013 | -0.008 | 0.129 | 0.138 |
| Cr | 0.829 | 0.827 | 0.469 | 0.467 | 0.005 | -0.005 | 0.164 | 0.165 |
| Cd | 0.875 | 0.876 | 0.386 | 0.398 | -0.0004 | -0.029 | 0.146 | 0.138 |
| Ni | 0.817 | 0.816 | 0.473 | 0.478 | 0.002 | 0.008 | 0.153 | 0.154 |
| Mean | 0.793 | 0.792 | 0.488 | 0.496 | 0.001 | -0.008 | 0.172 | 0.171 |
